# SupplAzithromycin for Prevention of Graft-Versus-Host Disease: A Randomized Placebo-Controlled Trial

**Published:** 2018-04-01

**Authors:** Maryam Barkhordar, Mehdi Mohammadi, Molouk Hadjibabaie, Ardeshir Ghavamzadeh

**Affiliations:** 1 Hematology-Oncology and Stem Cell Research Center, Tehran University of Medical Sciences, Tehran, Iran; 2 Department of Clinical Pharmacy, School of Pharmacy, Alborz University of Medical Sciences, Karaj, Iran; 3 Research Center for Rational Use of Drugs, Tehran University of Medical Sciences, Tehran, Iran; 4 Department of Clinical Pharmacy, School of Pharmacy, Tehran University of Medical Sciences, Tehran, Iran

**Keywords:** Azithromycin, Graft-versus-host disease, Mucositis

## Abstract

**Background: **Allogeneic hematopoietic stem cell transplantation has been used widely to treat various types of malignant and non-malignant disorders. Graft-versus-host disease is one of the main complications of this procedure which is associated with considerable mortality and affects quality of life. Despite careful selection of HLA-matched donors and implementing immunosuppressive therapy, the incidence rate of graft-versus-host disease remains high. Macrolide antibiotics are well-known immunomodulatory agents and have been effective as prophylaxis for graft-versus-host disease in preclinical studies.

**Materials and Methods:** Ninety-six adult patients with acute leukemia were recruited into a double-blind, randomized, placebo-controlled trial. All patients were first-time transplant candidates for a full-matched related or unrelated donor. Patients were allocated to receive azithromycin 500 mg daily (n=48) or placebo (n=48) from day -6 to +12. All patients received high-dose chemotherapy, standard immunosuppressive regimen and supportive care according to institutional protocols.

**Results:** The incidence of acute graft-versus-host disease grade III-IV and chronic graft-versus-host disease garde I-III was not significantly different between the two study arms. Oral mucositis grade 1-3 occurred in significantly lower number of patients in the azithromycin group compared with placebo.

**Conclusion:** Based on the results of this study, protective effect of azithromycin on graft-versus-host disease could not be demonstrated.

## Introduction

 Allogeneic hematopoietic stem cell transplantation has been the standard of care for various types of malignant and non-malignant disorders ^1^^-^^3^ . This procedure consists of high-dose chemotherapy, either myeloablative or nonmyeloablative, followed by transfusion of hematopoietic progenitor cells which repopulate the bone marrow with normal precursors.

Although transplantation can be a life-saving procedure, it may be complicated in some patients affected by treatment-related morbidities. These may include, but not limited to, serious infections, graft-versus-host disease (GvHD) and adverse effects of chemotherapeutic agents.

Disruption of intestinal mucosa in the course of conditioning regimen leads in translocation of endotoxins into circulation and cytokine release. Subsequent activation of T lymphocytes initiates a series of reactions, leading to widespread invasion of the host cells^        4 ^. Despite careful selection of HLA-matched donors and prophylactic use of immunosuppressive medications, GvHD remains a major contributor to post-transplantation morbidity and mortality^   5 ^.

Several studies have been conducted to find new agents for prophylaxis of acute GvHD 6^,^^7^ . Of note, the problem is not only to prevent GvHD but also to preserve the graft versus tumor effect at the same time. Moreover, the prophylactic agent must have limited interference with engraftment and be tolerable for the patient.

Immunomodulatory effects of macrolide class of antibiotics have been well described previously. A number of signaling pathways, including MAPK/NF-κB, have been implicated as the possible mechanisms of observed effects. The beneficial effects of macrolide antibiotics have been observed in various clinical settings including asthma, chronic obstructive pulmonary disease, cystic fibrosis, etc. Treatment with azithromycin led to significant improvement in FEV1 in patients affected with bronchiolitis obliterans syndrome, a manifestation of chronic GvHD of the lungs^8^. Azithromycin inhibited release of proinflammatory cytokines such as IL-8 and GM-CSF from bronchial epithelial cells after human lung transplantation^        9 ^. Based on data obtained from preclinical studies, prophylactic use of azithromycin in peri-transplantation period may be valuable for prevention of acute GvHD^10^. To the best of our knowledge, the effect of azithromycin on prevention of GvHD in humans has not been investigated yet. Therefore, the aim of this study was to find out whether adding azithromycin to conventional immunosuppressive therapy helps to prevent GvHD.

## MATERIALS AND METHODS

**Study design**

We conducted a double-blind, randomized, placebo-controlled trial at Hematology-Oncology and Stem Cell Research Center (HOSCRC) of Shariati Hospital, affiliated with Tehran University of Medical Sciences, Tehran, Iran. The trial was performed from September 2014 to March 2015. The study protocol was reviewed and confirmed by the Ethics Committee of the institution. All participants signed an informed consent form before enrollment (Trial registration ID: IRCT201403281030N16).

**Patients**

Ninety-six adult patients with AML or ALL were recruited into the study. All participants were candidates for first-time allogeneic stem cell transplantation from a full-match related or unrelated donor. Cardiac, pulmonary, renal, and hepatic function were normal according to the institutional protocol. Patients with Karnofsky performance status <70% or those who required treatment with azithromycin during the study period were excluded from the trial. 

**Intervention**

Patients were randomized to receive either azithromycin (n=48) or placebo (n=48) using a balanced block randomization model. The intervention group received one azithromycin 500 mg capsule P.O. daily (Tehran Chemie Pharmaceutical Co., Tehran, Iran) from 6 days before to 12 days after transplantation (days -6 to +12). The control group received placebo capsule with the same shape, size and color (Tehran Chemie Pharmaceutical Co., Tehran, Iran) on a similar schedule. High-dose chemotherapy was performed according to the institutional standard consisting of busulfan 1 mg/kg P.O. four times per day for a total of 16 doses, followed by cyclophosphamide 60 mg/kg i.v. daily for 2 consecutive days. Hematopoietic progenitor cells were collected peripherally, stored and infused 1 day after completion of chemotherapy. 

GvHD prophylaxis regimen consisted of cyclosporine and methotrexate. Cyclosporine was administered as i.v. infusion and in a dose of 1.5 mg/kg/d from day -3 to +8, followed by increasing the dose to 3 mg/kg/d until the patient tolerated oral intake. Afterwards, the drug was administered orally in a dose of 6 mg/kg/day. Dosage of methotrexate was 10 mg/m^2^ on day +1, and 6 mg/m^2^ on days +3, +6, and +11, which was administered as i.v. infusion. Additional prophylaxis with anti-thymocyte globulin (ATG) was considered for patients who received transplantation from unrelated donors. The dosage was 2.5 mg/kg/day i.v. from days -5 to -2.

All patients received similar supportive care for oral mucositis (OM). This included appropriate mouth cleansing and tooth brushing. Prophylactic medications included sucralfate 500 mg chewable tablet three times per day, nystatin oral suspension 20 drops every 3 hours and a mixture of 10 ml chlorhexidine 0.2% and 10 ml diluted povidone-iodine mouthwash administered every 3 hours. In case of severe pain, narcotic analgesics were used on an individual basis.

**Outcomes**

Primary study outcomes were incidence and severity of acute and chronic GvHD. Diagnosis and staging of GvHD were made according to internationally accepted criteria^        11 ^ and recorded by the evaluating investigator. Evaluations comprised of physical examinations and routine laboratory tests including liver enzymes, which were performed daily during hospital stay and periodically thereafter (i.e. weekly during the first 3 months; biweekly until month 6; and monthly until the end of first year after transplantation).

Secondary outcomes of the study consisted of incidence and severity of OM, incidence of CMV disease, relapse of primary disease and mortality.

Staging of OM was according to World Health Organization oral toxicity scale^   12 ^ (Grade 0: none; grade I: soreness ± erythema; grade II: erythema, ulcers, and patient can swallow solid diet; grade III: ulcers, extensive erythema, and patient cannot swallow solid diet; grade IV: mucositis to the extent that alimentation is not possible). Patients were assessed for OM daily from the day of transplantation until resolution of symptoms or day +21, whichever occurred earlier. All patients underwent a complete oral examination at baseline to detect and treat any existing source of infection or inflammation.

All clinical evaluations were performed by one investigator (M B) under supervision of an attending physician (A Gh). Patients, nursing staff, outcome assessor and attending physician were all blinded to allocations. 

Assessment for CMV infection was performed using viral pp65 antigen testing based on institutional protocol. Relapse of primary disease and mortality were also evaluated and recorded.

**Statistical analysis**

Pearson’s chi-squared test or Fisher’s exact test was used to compare categorical variables between the two groups. The Kaplan-Meier method and Log-rank test were performed to determine if there were differences in overall survival and relapse-free survival between groups. P-values less than 0.05 were considered statistically significant. Data analysis was performed using SPSS Statistics software (Version 19.0. IBM Corp. Armonk, NY). 

## Results

**Figure 1:**
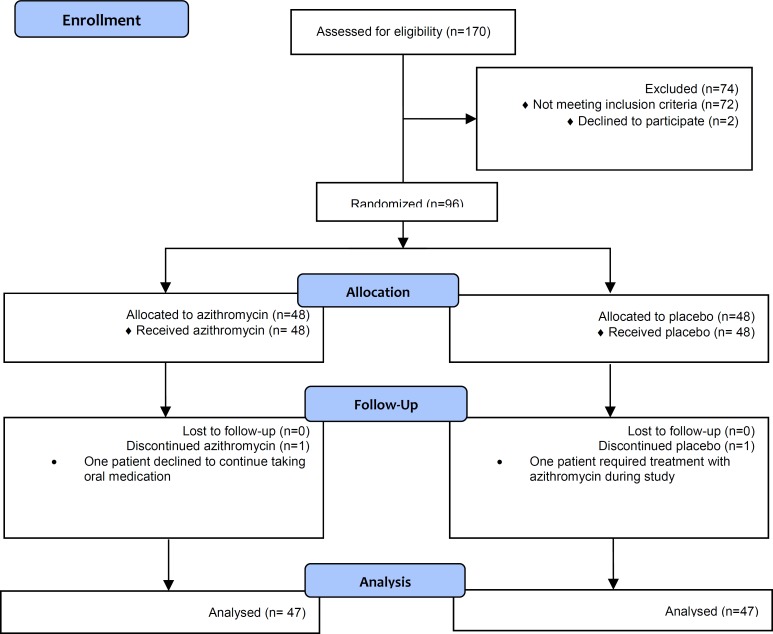
The Consort flow diagram of study

 Ninety-six patients were randomized into the study groups; 94 of whom completed the treatment course. The CONSORT flow diagram of progression through the study is presented in Figure 1.

Demographic characteristics and baseline variables were not significantly different between the two groups (Table 1).

**Table 1:** Baseline characteristics of participants

	**Azithromycin** **(n=47)**	**Control** **(n=47)**
Male (%)	32 (68.1%)	29 (61.7%)
Age (mean±SD, range)	35.5 ± 12.0 (16-62)	36.1 ± 11.5 (18-62)
Diseases		
AML	30 (63.8%)	31 (66.0%)
ALL	17 (36.2%)	16 (34.0%)
Donors		
Brother	32 (68.1%)	28 (59.7%)
Sister	12 (25.6%)	15 (31.9%)
Other related	0 (0.0%)	2 (4.2%)
Unrelated	3 (6.3%)	2 (4.2%)

There were no differences in the incidence of severe acute GvHD grade III-IV or the overall incidence of chronic GvHD grade I-III between the two study groups. Moreover, there was a trend toward lower incidence of grade II-III chronic GvHD in the intervention arm, but the difference was only marginally significant (34.0% vs. 48.9%, P=0.07; Table 2).

**Table 2:** Outcome of GvHD

	**Azithromycin**		**Control**		**p-** **value**
	**number**	**%**	**number**	%	
Acute GvHD grade 0-II	43	91.4	37	78.7	0.18
Acute GvHD grade III-IV	4	8.5	8	17.0	
Chronic GvHD grade I	25	53.2	16	34.0	0.07
Chronic GvHD grade II- III	16	34.0	23	48.9	

**Figure 2:**
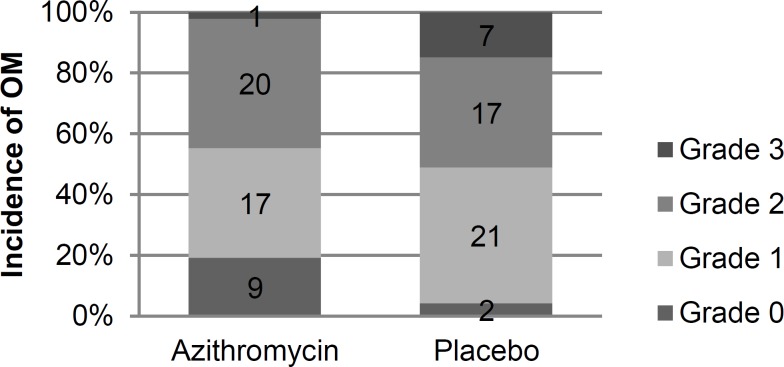
Frequency of OM ( oral Mucositis) according to WHO to WHO oral toxicity scale

None of the patients experienced grade 4 OM. The overall incidence of grade I-III OM was significantly lower in the intervention group compared with placebo (80.8% vs. 95.7%, P=0.02; Figure 2). 

The incidence of grade II-III OM was not different between the two groups (44.7% vs. 51.1%, *P*=0.68). None of the patients required treatment with narcotic analgesics.

CMV disease occurred in 25 patients of the intervention and 27 patients of the control group which was not significantly different (53.2% vs. 57.4%, respectively, *P*=0.68).

Engraftment of platelet and PMN in the intervention and control arm occurred after 12.5±1.6 vs. 12.2±1.5 days (p=0.27) and 13.3±1.8 vs. 13.7±1.8 days (p=0.28), respectively. Therefore, there was no significant difference in the time of engraftment between the two study arms.

**Figure 3:**
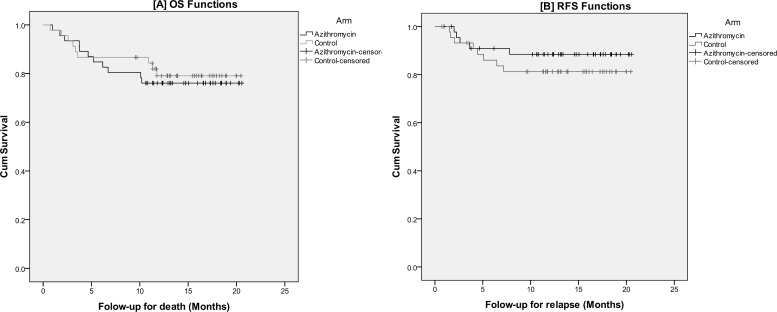
The Kaplan-Meier plots for overall survival and relapse-free survival

The median duration of follow-up for survivors was 15.67 months (range: 9.54-20.56). During this period, 14 patients experienced relapse of the primary disease (5 in azithromycin and 9 in the control group; *p*=0.22). Twenty-one deaths occurred during the study period; 11 in azithromycin and 10 in the control group. The reasons for death were relapse of primary disease in 12 (5 in azithromycin and 7 in the control group), infection in 6 (4 in azithromycin and 2 in the control group), GvHD in 1 (in the azithromycin arm), and other reasons in 2 (1 in each arm) patients. The Kaplan-Meier plots for the 1-year overall survival and 1-year relapse-free survival are presented in Figure 3. As shown, the survival distributions for the two arms were not significantly different [p=0.67 for 1-year overall survival; 88.5% vs. 81.2%, p=0.37 for 1-year relapse-free survival].The medication was well tolerated by all patients.

## Discussion

 The occurrence of GvHD after allogeneic stem cell transplantation is associated with substantial morbidity and mortality. In addition, the cost of the procedure is significantly increased^        13 ^. Because of the exhausting nature of the reaction and its substantial physical and psychological burden, prevention remains the mainstay of patient management. The most extensively used regimen for prevention of GvHD consists of a calcineurin inhibitor plus short-term methotrexate. Unfortunately, despite using this regimen, the incidence of GvHD remains high. Inhibition of host dendritic cell maturation may have additional benefits when combined with suppression of donor T lymphocytes. Dendritic cells are antigen-presenting cells which provide the infused T lymphocytes with host alloantigens. While the current immunosuppressive regimens used for prophylaxis of GvHD mainly target the action of T lymphocytes, azithromycin may exert additional benefits by suppressing the activation of dendritic cells through inhibition of NF-B. This mechanism of action has been demonstrated in a mouse model of GvHD^10^.

In general, the protective effect of azithromycinon on the incidence of GvHD could not be demonstrated based on the results of this trial. The only remarkable finding was a marginally significant lower incidence of severe chronic GvHD. Various immunosuppressive agents have been explored for use as prophylaxis of GvHD. Socié et al. conducted a long-term follow-up of 201 patients who received ATG in addition to the standard cyclosporine plus methotrexate^        14 ^. After 3 years, 12.2% of ATG-treated patients and 45.0% of the control patients developed extensive chronic GvHD (p<0.0001). In another study by Armand et al. , the addition of sirolimus to the standard regimen led to significant decrease in the incidence of acute GvHD grades II-IV (9% vs. 25%, p=0.015), but this was not associated with improved survival^        6 ^. It seems that, depending on the specific point of immune cascade which is affected by the immunosuppressant agent, various results will be anticipated. The optimal dosing and duration of treatment with azithromycin have not been determined yet. Future trials may focus on chronic GvHD as the clinical endpoint. Of note, the treatment did not interfere with engraftment and no negative effect on acute GvHD was observed.

The remarkable finding of the study was the lower incidence of OM in the intervention arm. Although the underlying mechanisms of protection have not been scrutinized yet, the role of NF-B cannot be overlooked. The release of NF-B in the second phase of OM development leads in up-regulation of gene transcription of pro-inflammatory cytokines such as TNF-, IL-1, and IL-6. This is followed by activation of some signaling pathways which terminate in the alteration of biologic activities of seemingly normal mucosa^        15 ^^.^ The suppressive effect of azithromycin on the production of NF-B has been well explained by previous studies. Aghai et al. obtained tracheal aspirate cells with activated NF-B from premature infants. TNF-mediated activation of NF-B was suppressed by the addition of azithromycin to the cell culture media. Moreover, production of IL-6 and IL-8 was also suppressed to the level of control group^        16 ^. Based on a NF-B assay study, the anti-inflammatory potency of azithromycin was 4 times weaker than that of hydrocortisone^   17 ^. Notably, macrolides are well distributed into tissue compartments including oral cavity. Azithromycin attained high concentrations in saliva, normal gingival and pathological periodontal tissue^        18 ^. The established immunomodulatory effects combined with adequate penetration into gingival tissue give explanations for the observed results. Additional trials are required to further delineate potential therapeutic benefits of azithromycin in OM.

There was no significant difference between the two arms regarding occurrence of CMV disease, relapse of primary disease or death. Noteworthy, the study was not adequately powered to detect any difference if exists. 

## CONCLUSION

 In general, the results of this study could not demonstrate a protective effect of azithromycin on the incidence of acute or chronic GvHD, but the observed effect of azithromycin on prevention of OM warrants further research. 
